# Comparative Analysis of Surface Layer Glycoproteins and Genes Involved in Protein Glycosylation in the Genus *Haloferax*

**DOI:** 10.3390/genes9030172

**Published:** 2018-03-20

**Authors:** Yarden Shalev, Shannon M. Soucy, R. Thane Papke, J. Peter Gogarten, Jerry Eichler, Uri Gophna

**Affiliations:** 1 School of Molecular and Cell Biology and Biotechnology, George S. Wise Faculty of Life Sciences, Tel Aviv University, Tel Aviv 69978, Israel; yardenshalev@mail.tau.ac.il; 2 Department of Molecular and Cell Biology, University of Connecticut, Storrs, CT 06269, USA; shannon.soucy@uconn.edu (S.M.S.); thane@uconn.edu (T.P.); 3 Institute for Systems Genomics, University of Connecticut, Storrs, CT 06269, USA; gogarten@uconn.edu; 4 Department of Life Sciences, Ben Gurion University of the Negev, Beersheva 8410501, Israel; jeichler@bgu.ac.il

**Keywords:** glycosylation, halophilic archaea, horizontal gene transfer, lateral gene transfer, mating, recombination, S-layer

## Abstract

Within the *Haloferax* genus, both the surface (S)-layer protein, and the glycans that can decorate it, vary between species, which can potentially result in many different surface types, analogous to bacterial serotypes. This variation may mediate phenotypes, such as sensitivity to different viruses and mating preferences. Here, we describe S-layer glycoproteins found in multiple *Haloferax* strains and perform comparative genomics analyses of major and alternative glycosylation clusters of isolates from two coastal sites. We analyze the phylogeny of individual glycosylation genes and demonstrate that while the major glycosylation cluster tends to be conserved among closely related strains, the alternative cluster is highly variable. Thus, geographically- and genetically-related strains may exhibit diverse surface structures to such an extent that no two isolates present an identical surface profile.

## 1. Introduction

The prokaryotic cell envelope provides archaeal and bacterial cells with a protective coat that resists environmental stressors both abiotic and biotic—namely viruses and toxins secreted by competing cells. In archaea, the surface layer (S-layer) is generally composed of a single protein that is always glycosylated [1]. *N*-glycosylation—the covalent attachment of glycans to select target asparagine residues in proteins—is thought to be one of the most common post-translational modifications of archaeal cell surface proteins, including S-layer glycoproteins, as well as flagelins [2] and pilins [3]. Archaeal *N*-linked glycans are richly diverse in composition and structure [4,5] and accordingly, archaeal *N*-glycosylation gene pathways show considerable variation in gene content, sharing only a few common components.

The *N*-glycosylation pathways of the halophilic archaeon *Haloferax volcanii* have been intensively studied and are relatively well-understood. *H. volcanii* contains two *N*-glycosylation pathways—a major pathway which is functional at all concentrations of salt tested and an alternative glycosylation pathway which is only recruited at lower salinity—with each pathway generating its own distinct glycan modification [6]. When relying on the general *N*-glycosylation pathway, the *H. volcanii* S-layer glycoprotein is modified by a pentasaccharide in a process whereby the glycosyltransferases AglJ, AglG, AglI and AlgE sequentially add the first four sugars of the *N*-linked pentasaccharide to a common dolichol phosphate carrier [7,8,9,10]. After the lipid-linked tetrasaccharide has been translocated across the membrane, the glycan is delivered to selected S-layer glycoprotein Asn-residues by AglB, the archaeal oligosaccharyltransferase [11,12]. The final sugar of the pentasaccharide is added to a distinct dolichol phosphate carrier on the cytoplasmic face of the membrane by the glycosyltransferase AglD [7,9]. The dolichol phosphate-bound sugar is then delivered across the membrane to face the exterior of the cell in a process involving AglR, at which point the sugar is transferred to the Asn-linked tetrasaccharide by AglS [9,13,14,15,16]. Other Agl proteins serve various sugar-processing roles that contribute to pentasaccharide assembly. AglF is a glucose-1-phosphate uridyltransferase, AglM is a UDPglucose dehydrogenase, AglP is a methyltransferase and AglQ is an isomerase [8,17,18,19]. All the genes encoding proteins known to participate in the assembly and attachment of a pentasaccharide to selected Asn residues of *N*-glycosylated proteins in *H. volcanii* are located within the same gene cluster beginning at the *aglJ* gene and extending to *aglM*, with the exception of *aglD* that is found outside this cluster [20].

The proteins responsible for assembly of the pentasaccharide *N*-linked glycan are not involved in the biosynthesis of the low salt glycan, a tetrasaccharide of distinct composition. *agl5*–*agl15* have been identified as comprising an alternative *N*-glycosylation pathway. Agl5 and Agl6 are implicated, in addition to a hexose to dolichol phosphate, while Agl7 contributes to the sulfation of this lipid-linked sugar. Agl8 and Agl9 are involved in the addition of a hexose to disaccharide-charged dolichol phosphate. Agl10–14 are involved in the subsequent addition of a rhamnose to the dolichol phosphate-bound trisaccharide, while Agl15 is predicted to serve as a flippase, mediating the translocation of the low salt tetrasaccharide-charged dolichol phosphate across the membrane [21].

Given that archaeal *N*-linked glycans can vary greatly in composition and structure between species and that different species within the same genus can have different glycosylation clusters [22], it is highly likely that in the environment, different, closely-related haloarchaeal strains may present a wide assortment of different surface decorations that can modify the different S-layer glycoproteins in each species. Here, we performed genomics analysis on multiple *Haloferax* strains isolated from two relatively close coastal sites by comparing their genomes to those of previously isolated named *Haloferax* species to investigate the diversity and evolution of genes that shape the surface of haloarchaeal cells. We used multiple phylogenetic analyses to show that while the S-layer glycoprotein appears to be mostly vertically inherited, lateral gene transfer can impact glycosylation genes even when gene organization is highly conserved. We also identified a novel major S-layer glycoprotein and validated it biochemically.

## 2. Materials and Methods

### 2.1. Sample Collection and 16S rRNA/polB Gene Sequencing

Stagnant seawater or dry salt samples were collected from four tidal/spray pools at two rocky shores along Israel’s Mediterranean coastline (Atlit (32°42′37.3″ N 34°56′32.0″ E) and Michmoret (32°24′23.8″ N 34°52′05.2″ E)). Five milliliters of seawater from each pool were spread onto a yeast extract, peptone and casmino acids (YPC) plate containing ampicillin and incubated at 42 °C until colonies appeared. Dry salt samples were dissolved in sterile seawater before spreading on plates. Colonies with typical *Haloferax* coloring and shape were further examined with 16S rRNA gene PCR using Halobacteriales-specific 16S rRNA gene primers 287F and 958R [23]. The <600 bp amplicon was sequenced by ABI 3730XL sequencers at MCLAB DNA Sequencing Services (San Francisco, CA, USA). Colonies that proved to belong to the *Haloferax* genus had their whole genome sequenced using Illumina MiSeq (Illumina, San Diego, CA, USA) 2 × 250 paired-end reads at the Weizmann Institute genomics facility (Weizmann Institute of Science, Rehovot, Israel). Raw reads were first trimmed with Cutadapt v1.9.1 [24] to remove adaptor sequences and bases with a Phred score lower than 20. Genomes were assembled using SPAdes v3.7.0 [25] with kmer sizes 21, 33, 55, 77, 99 and 127. Assembly quality was checked using Quast v2.3 [26] and coding sequences and annotations were predicted using Prokka v1.11 [27], ignoring contigs that were shorter than 200 base pairs.

### 2.2. Genomic DNA Extraction by Spooling

Cultures of *Haloferax* colonies were grown to 2.0 OD_600_ under shaking conditions at 45 °C for two nights. 1.5 mL of culture was centrifuged for 5 min at 6000× *g*, then resuspended in 200 μL of ST buffer (1 M NaCl, 20 mM Tris-HCl) and 200 μL of lysis solution (100 mM EDTA pH 8.0, 0.2% SDS) is added. 1 mL of ethanol was added and the DNA spooled onto a glass capillary until liquid was homogeneous and clear. The DNA was washed 3 times by transferring the spooled DNA to a microcentrifuge tube with 1 mL of fresh ethanol and was then allowed to dry. The DNA was then solubilized in 250 μL Tris EDTA (TE) buffer.

### 2.3. Phylogenetic Analysis

The protein and nucleotide sequences of AglB and RpoB1 from *H. volcanii* DS2 and *H. Gibbonsii* ATCC 33959 were retrieved from the Kyoto Encyclopedia of Genes and Genomes (KEGG) database [28]. The protein and nucleotide sequences of AglB and RpoB1 from *H. lucentense* DSM 14919 and *H. Denitrificans* ATCC 35960 were retrieved from the National Center for Biotechnology Information (NCBI) database. Gene and protein sequences of the *Haloferax* isolates were aligned using MAFFT [29]. Trees were constructed using maximum-likelihood as implemented in MEGA7 [30], with 100 bootstrap trials. The evolutionary history was inferred from the nucleotide sequences using the Maximum Likelihood method, based on the Tamura-Nei model [31], and in the case of the protein sequence AglB, the evolutionary history was inferred by using the Maximum Likelihood method based on the JTT matrix-based model [32]. The tree with the highest log likelihood is shown in all phylogeny figures. Initial tree(s) for heuristic searches were obtained automatically by applying Neighbor-Join and BioNJ algorithms to a matrix of pairwise distances, estimated using the Maximum Composite Likelihood (MCL) approach and then by selecting the topology with the superior log likelihood value.

### 2.4. Estimation of the Significance of a Protein Sequence Alignment Using PRSS

PRSS (protein sequence alignment significance) analyses was performed to assess homology through shuffling from pairwise sequence comparison [33,34].

### 2.5. Protein Function Prediction

Protein function predictions of genes in glycosylation clusters were performed with HHpred [35].

### 2.6. Recombination Analysis

Recombination analysis was performed using the Recombination Detection Program (RDP) v4, which uses multiple detection algorithms including the RDP Method [36], GENECOV [37], BootScan [38], MaxChi [39], Chimaera [40], SiScan [41] and 3Seq [42] to check for the presence of recombinant sequences [43]. Events were considered reliable only if at least four of these algorithms supported recombination.

### 2.7. Average Nucleotide Identity Calculation

The average nucleotide identity (ANI) calculator used [44] estimated the average nucleotide identity using reciprocal best hits (two-way ANI) between two genomic datasets, as calculated in [45].

### 2.8. Culture Conditions

*H. volcanii* cells were routinely grown (as described in Reference [46]) in a rich medium (Hv-YPC) containing (per liter) 144 g of NaCl, 21 g of MgSO_4_·7H_2_O, 18 g of MgCl_2_·6H_2_O, 4.2 g of KCl and 12 mM Tris-HCl (pH 7.5). For solid media, agar (Difco, BD, NJ, USA) was added at a concentration of 15 g per liter and was dissolved by heating the medium on a hot plate. Yeast extract (0.5%, wt/vol; Difco), peptone (Bacto; 0.1%, wt/vol) and casamino acids (Difco; 0.1%, wt/vol) were added and the medium was autoclaved. After cooling, CaCl_2_ was added to a final concentration of 3 mM.

### 2.9. Protein Sample Preparation

Cultures of *H. volcanii* cells were grown to 1.0 OD_600_ under shaking conditions at 45 °C overnight. To 100 µL aliquots, 43 µL of 50% Trichloroacetic acid (15% final concentration) were added. Samples were incubated for 30 min on ice and then centrifuged at 10,000× *g* for 15 min at 4 °C. One mL of ice-cold acetone was added to the pellet, which was then centrifuged again at 10,000× *g* for 15 min at 4 °C. The pellet was left to dry for 5 min, after which time 30 µL of 2× sample buffer was added. The samples underwent sonication for 1 min in a supersonic cleaner (model DG-1, M.R.C, Holon, Israel), heated for 5 min at 95 °C and then separated by 6% SDS-PAGE.

### 2.10. SDS-PAGE and Protein Detection

SDS-PAGE was performed as described [47]. The gel was fixed in staining solution (0.1% Coomassie Brilliant Blue R-250, 50% methanol and 10% acetic acid) for from an hour to overnight with gentle agitation, followed by destaining in 40% methanol and 10% acetic acid.

### 2.11. Mass Spectrometry

Mass spectrometry was performed by the Smoler Proteomics Center at the Technion, Israel Institute of Technology. Samples were digested by chymotrypsin and analyzed by LC-MS/MS on a Q-Exactive (Thermo, Waltham, MA, USA) apparatus. Samples were identified using Discoverer 1.4 with the Sequest search algorithm (Thermo, Waltham, MA, USA).

## 3. Results

### 3.1. Phylogenetic Analysis of the Isolates Using the rpoB1 Gene and ANI

**Figure 1:**
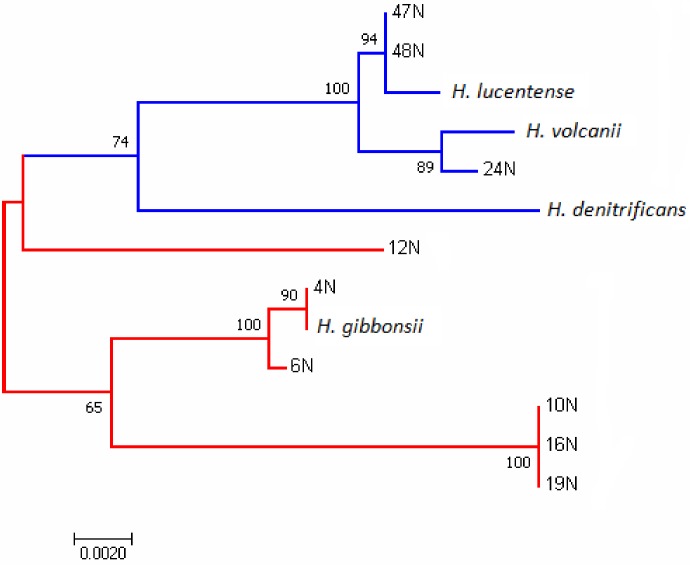
Molecular phylogenetic analysis of the *rpoB1* gene by Maximum Likelihood. Red lines represent species and isolates with an S-layer glycoprotein corresponding to that of *H. gibbonsii*. Blue lines represent the species and isolates with an S-layer glycoprotein corresponding to the *H. volcanii* S-layer glycoprotein (see below Section 3.2). The evolutionary history was inferred by using the Maximum Likelihood method based on the Tamura-Nei model [31]. The tree with the highest log likelihood (−3138.94) is shown. The percentage of trees in which the associated taxa clustered together is shown next to the branches. A discrete Gamma distribution was used to model evolutionary rate differences among sites (5 categories (+G, optimized shape parameter = 0.0500)). The rate variation model allowed for some sites to be evolutionarily invariable ((+I), 47.73% sites). The unrooted tree is drawn to scale, with branch lengths measured in the number of substitutions per site. The analysis involved 13 nucleotide sequences. Codon positions included were 1st + 2nd + 3rd. All positions containing gaps and missing data were eliminated. A total of 1827 positions were in the final dataset. Evolutionary analyses were conducted in MEGA7 [30].

**Figure 2:**
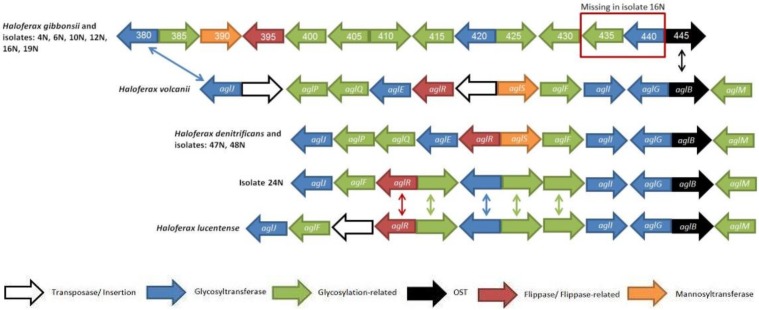
Schematic presentation of the *aglB*-based glycosylation clusters of four different *Haloferax* strains and the nine *Haloferax* isolates. The sizes of the genes are arbitrary. Arrows in between clusters indicate homology. The legend describes gene function according to the color scheme at the bottom. Protein function prediction was performed using HHpred [35]. Based on Figure 2 from Ref. [22].

Colonies with typical *Haloferax* morphology isolated from samples of stagnant seawater or dry salt samples from two rocky shores along Israel’s Mediterranean coastline (Atlit and Michmoret) were further examined and subsequently the strains giving rise to these colonies, had their genomes sequenced (Table 1, see Section 2). The 16S rRNA gene of all these isolates had over 99% identity to that of the *H. volcanii* type strain and consequently was deemed too conserved for resolving relatedness between isolates. Therefore, to determine the phylogenetic relationship between isolates, we used the *rpoB1* gene (an RNA polymerase subunit encoded by conserved single-copy gene), which serves as a suitable phylogenetic marker for Halobacteriales [48]. The *rpoB1* gene of *H. gibbonsii* was added to the analysis since isolates 4N, 6N, 10N, 12N, 16N and 19N (henceforth the *H. gibbonsii* clade) showed the highest sequence similarity to *H. gibbonsii rpoB1*. The *rpoB1* tree (Figure 1) clustered into four main groups; isolates 4N and 6N clustered with *H. gibbonsii*, while 12N was deep-branching. Isolates 10N, 16N and 19N grouped together and had identical *rpoB1* sequences, whereas the rest of the isolates grouped with *H. volcanii* and *H. lucentense*. Isolates 24N, 47N and 48N resembled *H. lucentense* and *H. denitrificans,* in terms of their main glycosylation pathway genes (see below, Figure 2) and therefore these species were added to the phylogenetic analysis. 

Haloarchaea have been shown to be highly recombinogenic in nature [49,50] and most transferred genes are incorporated into recipient genomes through homologous recombination [51]. Thus, single gene phylogenies should be accompanied by recombination detection. No recombination events between these *rpoB1* sequences could be detected in the rpoB1 gene nucleotide sequence (Figure S1), possibly because this gene is not sufficiently variable to allow reliable detection. To avoid relying on a single gene marker for inference of genetic relatedness with an analysis of average nucleotide identity (ANI) of shared genes in these genomes [52] was performed. The ANI data were congruent with the *rpoB1* results (Table S1), with 4N and 6N showing higher sequence similarity to *H. gibbonsi* than did isolates 10N, 16N and 19N and 12N, which corresponded to a more distant lineage. In contrast to the *rpoB1* phylogeny, 24N appears to be closely related to *H. volcanii*, yet shares higher average nucleotide identity to *H. lucentense*.

### 3.2. Two Distinct S-layer Glycoproteins Exist within the Closely-Related Haloferax Isolates

**Figure 3:**
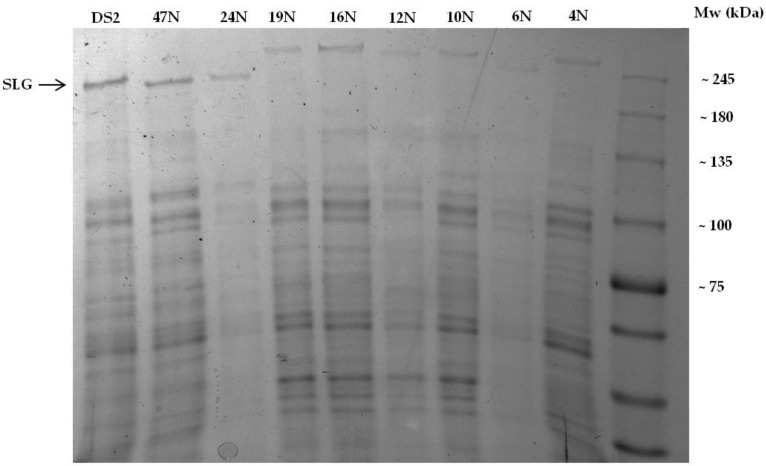
SDS-PAGE gels of protein samples extracted from the isolates and *H. volcanii.* The putative S-layer glycoprotein of 48N is identical to that of 47N (Figure S7).

The S-layer glycoprotein serves as the sole component of the protein layer surrounding *H. volcanii* cells and migrates on SDS-PAGE like a 205 kDa polypeptide [53]. Even though all the isolates collected along Israel’s Mediterranean coastline belonged to closely-related *Haloferax* lineages (see above), the strong high molecular weight SDS-PAGE bands representing putative S-layer glycoproteins in the isolates belonging to the *H. gibonsii* clade, as assigned on the basis of their *rpoB* phylogeny (4–19N), migrated slower than did their *H. volcanii* clade counterparts (including isolates 24–48N, Figure 1) suggesting differences in the sequences of their S-layer glycoproteins that may vary in molecular weight but also in their acidic amino acid content. We identified using InterPro [54] proteins from available haloarchaeal genomes that belonged to the IPR026458 family, having both a surface glycoprotein signal peptide (IPR026452) and a PGF-CTERM domain and an archaeal protein-sorting signal (IPR026371) (a C-terminal tripartite structure including a highly conserved proline-glycine-phenylalanine (PGF) motif). These proteins were then used to query the isolate genomes predicted proteins using BLAST [55]. As expected, the protein sequence acquired from *H. gibbonsii* (accession A0A0K1IRS6) had homologous matches in all the isolates from the *H. gibbonsii* clade but not to the S-layer glycoprotein present in *H. volcanii* DS2. The molecular weight of the predicted *H. gibbonsii* clade S-layer glycoprotein without covalently linked glycans was calculated to be 89.8 kDa [56], which is similar to the molecular weight that of its *H. volcanii* counterpart, which is 85.2 kDa [42]. Following gel extraction of the predicted S-layer glycoprotein band (Figure 3) and chymotrypsin digestion of the protein, mass spectrometry confirmed that the slower migrating band of the *H. gibbonsii* clade indeed corresponded to A0A0K1IRS6, verifying that this is indeed a novel *Haloferax* S-layer glycoprotein. The fact that the two separate clades based on *rpoB1* phylogeny express two distinct types of S-layer glycoproteins (Figure 1) implies that the genes encoding these proteins were not horizontally transferred between these clades [53] but rather that one of the clades has probably acquired its ancestral protein from a more distant lineage. A0A0K1IRS6 from *H. gibbonsii* and AAA72996.1 from *H. volcanii* showed only insignificant matches in the carboxy terminal part of the *H. volcanii* S-layer protein using BLASTP [55]; however, comparing the whole sequences in PRSS [33] revealed significant sequence similarity (using a PAM 400 matrix resulted in a *z*-value of 181 and an E(1) value of 2.7 × 10^−8^). This suggests that the two types of S-layer proteins are divergent homologs. Recombination analysis of the two types of S-layer glycoproteins separately showed that both the *H. gibbonsii* clade and the *H. volcanii* clade underwent at least three recombination events each (Figures S2 and S3).

### 3.3. The Composition of Genes in N-Glycosylation Pathway Clusters Varies in the Isolates

The isolates collected along Israel’s Mediterranean coastline were sequenced and glycosylation gene clusters were identified in BLASTX searches [57]. In Archaea, the gene encoding the oligosaccharyltransferase AglB is almost universally detected and, in many cases, is found as part of a cluster of putative *N*-glycosylation genes [22]. *H. volcanii* contains the most studied and best-understood glycosylation pathway in Archaea. Based on what is known in *H. volcanii* [20], glycosylation clusters in the isolates were defined as including those genes between the *aglB* and *aglJ* homologs (encoding the glycosyltransferase responsible for adding the first sugar of the *N*-linked pentasaccharide [58]). The *aglJ* gene was chosen as the border of the glycosylation cluster since it is the last gene in the *H. volcanii* glycosylation cluster and since *aglJ* homologs were present in all the isolate clusters. Indeed, no ORFs with sequence similarity to any known glycosylation protein were observed upstream to aglJ in any of the isolate genomes.

As seen in Figure 2, most isolate glycosylation gene clusters (six out of nine) were found to be identical to that of *H. gibbonsii*, with only 16N missing two glycosylation genes. Two other isolates, 47N and 48N, are similar to the *H. volcanii* and putative *H. denitrificans* glycosylation gene clusters (the two species contain homologous genes in the same order and orientation, other than two transposable element insertions found in the *H. volcanii* cluster). The glycosylation gene cluster in isolate 24N is similar to that in *H. lucentense*, with these genomes sharing more homologous genes with *H. volcanii* and *H. denitrificans* than with *H. gibbonsii*.

Even though the isolates belonging to the H. gibbonsii clade share a similar glycosylation gene cluster with *H. gibbonsii*, they group separately in terms of their rpoB1 phylogeny (Figure 1). Likewise, 24N has a glycosylation gene cluster similar to that of *H. lucentense*, yet groups closer to *H. volcanii* in the evolutionary analysis than did isolates 47N and 48N, which group closer to *H. lucentense*, even though their glycosylation gene cluster is similar to that of *H. volcanii* (Figure 1).

Our results indicate that isolates and species that are similar in their glycosylation gene cluster pattern also share a type of S-layer glycoprotein. *H. gibbonsii* and the isolates that belong to the *H. gibbonsii* clade all express the same type of S-layer glycoprotein and share highly similar glycosylation gene clusters. Isolates 47N and 48N present glycosylation gene clusters similar to that of *H. volcanii* and share the type of S-layer glycoprotein. Although 24N and *H. lucentense* do not contain a glycosylation gene cluster identical to that *H. volcanii*, they do share homologous glycosyltransferases and other glycosylation-related genes, as well clustering on the *rpoB1* tree (Figure 1). This implies that within these related lineages, major glycosylation gene clusters have been mostly vertically inherited, despite the fact that glycosylation genes in *Haloferax* have been shown to be frequently horizontally transferred [22].

### 3.4. The Glycosyltransferase Gene aglJ Is Relatively Conserved

**Figure 4:**
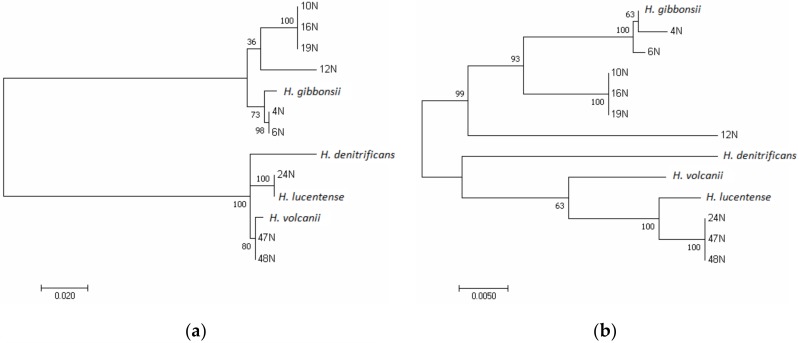

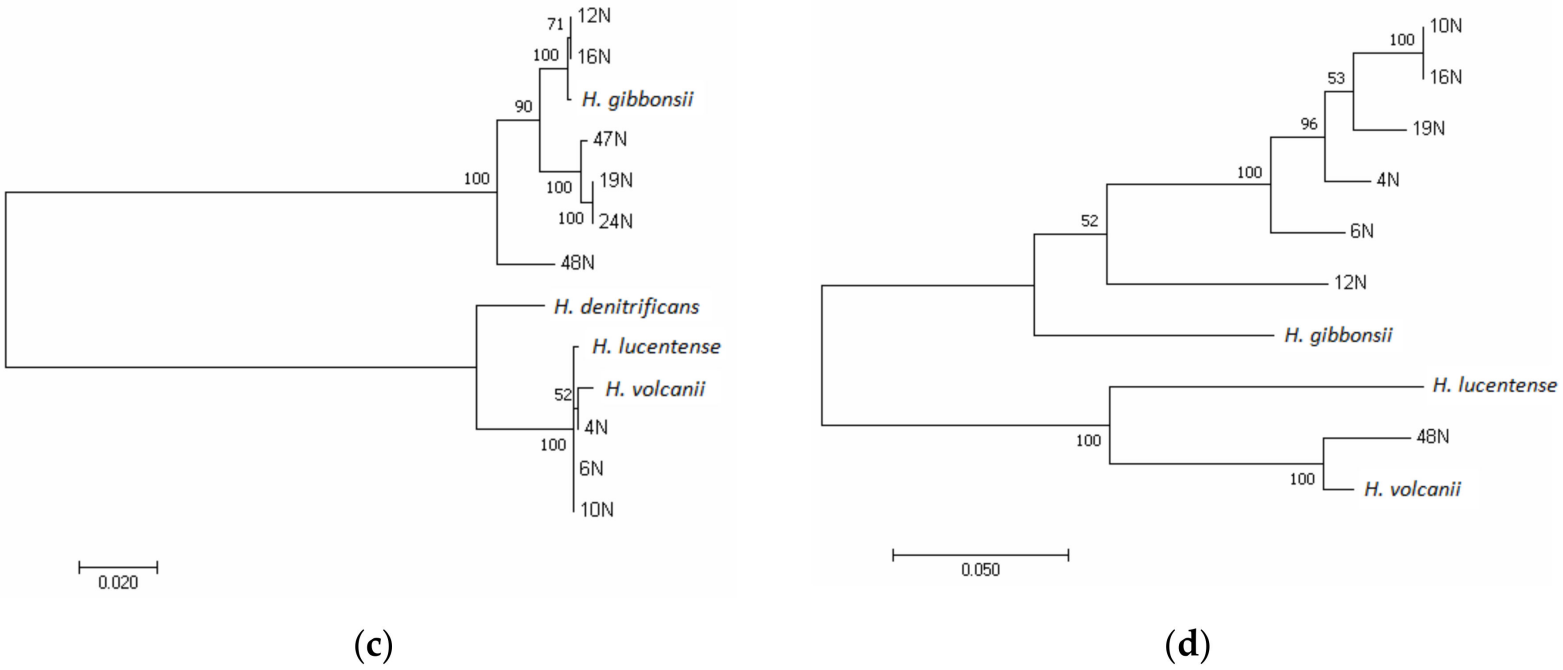
Molecular phylogenetic analysis by Maximum Likelihood method of genes related to glycosylation. The evolutionary history of trees (**a**,**b**,**d**) were inferred by using the Maximum Likelihood method based on the Tamura-Nei model [31]. The percentage of trees in which the associated taxa clustered together is shown next to the branches. The unrooted trees are drawn to scale, with branch lengths measured in the number of substitutions per site. Evolutionary analyses were conducted in MEGA7 [30]. (**a**) Molecular phylogenetic analysis by Maximum Likelihood method of glycosyltransferase-encoding *aglJ* gene. The tree with the highest log likelihood (−2139.14) is shown. A discrete Gamma distribution was used to model evolutionary rate differences among sites (5 categories (+G, optimized shape parameter = 0.1945)). The rate variation model allowed for some sites to be evolutionarily invariable ((+I), 41.18% sites). The analysis involved 13 nucleotide sequences. Codon positions included were 1st + 2nd + 3rd. All positions containing gaps and missing data were eliminated. A total of 901 positions were in the final dataset. (**b**) Molecular phylogenetic analysis by Maximum Likelihood method of mannosyltransferase-encoding *aglD*. The tree with the highest log likelihood (−3615.77) is shown. A discrete Gamma distribution was used to model evolutionary rate differences among sites (5 categories (+G, optimized shape parameter = 0.1525)). The rate variation model allowed for some sites to be evolutionarily invariable ((+I), 53.21% sites). The analysis involved 13 nucleotide sequences. Codon positions included were 1st + 2nd + 3rd. All positions containing gaps and missing data were eliminated. A total of 1875 positions were in the final dataset. (**c**) Molecular phylogenetic analysis by Maximum Likelihood method of the oligosaccharyltransferase AglB. The evolutionary history was inferred by using the Maximum Likelihood method based on the JTT matrix-based model [32]. The tree with the highest log likelihood (−4643.58) is shown. A discrete Gamma distribution was used to model evolutionary rate differences among sites (5 categories (+G, parameter = 200.0000)). The rate variation model allowed for some sites to be evolutionarily invariable ((+I), 33.98% sites). The analysis involved 13 amino acid sequences. All positions containing gaps and missing data were eliminated. A total of 1040 positions were in the final dataset. (**d**) Molecular phylogenetic analysis by Maximum Likelihood method of the glycosyltransferase-encoding *agl6*. The tree with the highest log likelihood (−4223.78) is shown. A discrete Gamma distribution was used to model evolutionary rate differences among sites (5 categories (+G, optimized shape parameter = 0.9222)). The rate variation model allowed for some sites to be evolutionarily invariable ((+I), 52.30% sites). The analysis involved 10 nucleotide sequences. Codon positions included were 1st + 2nd + 3rd. All positions containing gaps and missing data were eliminated. A total of 1242 positions were in the final dataset.

The gene encoding glycosyltransferase AglJ, which is located on the opposite end of that encoding AglB in the *H. volcanii* glycosylation gene cluster, has homologs in the genomes of all of the isolates and the related species examined. AglJ adds the first sugar of the *N*-linked pentasaccharide decorating the *H. volcanii* S-layer glycoprotein [46] and should therefore be sugar-specific. Overall, the phylogenetic tree of *aglJ* (Figure 4a) resembles the tree constructed from the conserved gene *rpoB1* (Figure 1)*,* with the only difference a switching of position between *H. volcanii* and *H. lucentense*, likely reflecting gene transfer within this group of closely related organisms. We analyzed the *aglJ* nucleotide sequence for within-gene recombination events and only one such event was found in all six sequences from the *H. volcanii* clade, where the recombined sequence originated in the *H. gibbonsii* clade (Figure S4). Thus, *aglJ* shows a predominantly vertical phylogenetic signal, yet even in this gene there is evidence of exchange between lineages.

### 3.5. Phylogeny of the Mannosyltransferase-Encoding aglD Gene Is Similar to aglJ in the H. gibbonsii Clade but Not in the H. volcanii-Related Isolates

The final sugar of the *N*-linked pentasaccharide decorating the *H. volcanii* S-layer glycoprotein is mannose, which is added to a dolichol phosphate carrier on the cytoplasmic face of the membrane by the glycosyltransferase AglD [11]. Contrary to other genes whose products participate in the assembly and attachment of the pentasaccharide to selected Asn residues of *N*-glycosylated proteins in *H. volcanii*, *aglD* is not located within the major glycosylation cluster and generally has higher G + C content than the rest of the genome (Table S2). Evolutionary analysis of *aglD* (Figure 4b) revealed that within the *H. gibbonsii* clade, the grouping was as observed for *aglJ*. However, in the *H. volcanii* clade, this was not the case. Here, isolate 24N grouped closer to isolates 48N and 47N, while *H. volcanii* occupied a more basal position than that observed in the *aglJ* and *rpoB1* trees. Thus, the location of a gene within or outside the glycosylation gene cluster does not predict its evolutionary history, which is not surprising as most transferred genes in haloarchaea are integrated into the recipient genome by way of homologous recombination [51]. Analysis of the *aglD* nucleotide sequence for within-gene recombination events revealed only one such event, which was found in *H. volcanii,* coming from within the *H. volcanii* clade (Figure S5).

### 3.6. The Gene Encoding AglB Most Likely Underwent Lateral Gene Transfer in Haloferax Species

AglB orthologues from different haloarchaea were shown to be able to functionally complement the *H. volcanii* enzyme and successfully transfer the tetrasaccharide precursor of the *N*-linked pentasaccharide decorating the *H. volcanii* S-layer glycoprotein from the dolichol phosphate carrier to target asparagine residues [59] and inter-species complementation was also successfully shown in methanogens [60]. This implies considerable substrate flexibility, which can theoretically allow an *aglB* homolog acquired by horizontal gene transfer (HGT) to replace an ancestral orthologue, in a process known as xenologous gene displacement [61]—the most frequent outcome of HGT in haloarchaea [51]. The phylogenetic tree constructed from the AglB protein sequence (Figure 4c) differs significantly from the phylogeny of the more conserved *rpoB1* shown in Figure 1. In contrast to *rpoB1*, the AglB tree forms two main groups. Moreover, the branching of the clusters and species branching as sister taxa in the AglB tree differs from the *rpoB1* tree, implying HGT. The gene encoding AglB is almost universally detected [22], yet there is substantial variation in the composition of *aglB*-proximal glycosylation gene clusters in the different *Haloferax* strains and species, even in those closely related, possibly also reflecting HGT. The *aglB*-based *N*-glycosylation pathways have been shown to have an active role in cell–cell interactions and quite possibly cell recognition and to be important in haloarchaeal mating, as well as in other S-layer-related functions [62].

Analysis of the *aglB* nucleotide sequence for within-gene recombination events detected two events within the *H. gibbonsii* clade (Figure S6), one of which involves a lineage outside the *H. gibbonsii* clade. These data further support a history of frequent transfer for this gene family, likely aided by its functional flexibility.

### 3.7. Alternative Glycosylation Clusters in Isolates and Related Haloferax Species

**Figure 5:**
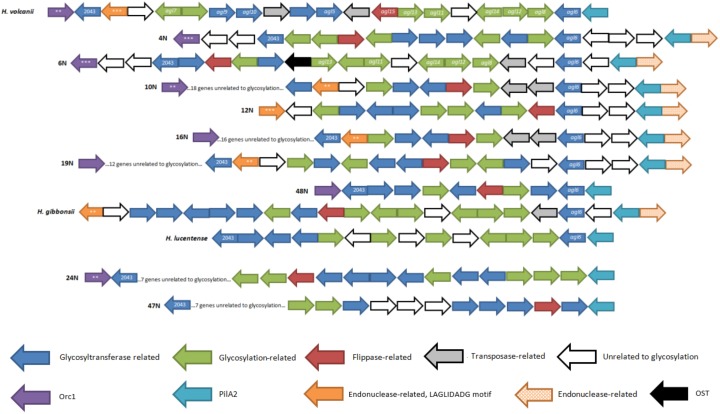
Schematic presentation of the *agl6*-based glycosylation gene clusters in four different *Haloferax* strains and the nine *Haloferax* isolates. The sizes of the genes are arbitrary. Asterisks indicate homology. Glycotransferases marked 2043 are homologs to *HVO_2043,* which is an uncharacterized gene adjacent to the alternative glycosylation gene pathway in *H. volcanii*. The legend describes the function assigned to each color used in the scheme. Protein function prediction was performed using HHpred [35]. Based on Figure 2 from Ref. [22].

It has been shown that *H. volcanii* responds to changes in environmental salinity by modulating the *N*-linked glycans decorating the S-layer glycoprotein [6], with a second *N*-glycosylation pathway being recruited in cells grown at lower salt concentrations [19]. Expression of these alternative glycosylation pathway genes results in a glycan chain that is different in composition than that produced by the main glycosylation pathway. The alternative glycosylation pathway in *H. volcanii* is composed of genes *agl5—agl15* which have been shown to participate in the generation of the low-salt tetrasaccharide [21]. Using BLASTX [57], we found that most of the genes comprising the alternative pathway in *H. volcanii* are absent in the isolates. We compared putative alternative glycosylation clusters in the *Haloferax* genomes described above, based on the presence of the hallmark gene *agl6* [21], which is present in all isolates except 24N and 47N. From preliminary analysis we discovered that in all the isolate clusters there was an adjacent *pilA2* gene downstream to *agl6*. PilA2 was conserved in all of the isolates, including 24N and 47N, which upon further examination also had multiple glycosylation and flippase-related genes adjacent to *pilA2*, as in the rest of the isolates. In contrast to the major glycosylation pathway (Figure 2), the alternative glycosylation clusters were highly variable across the different genomes (Figure 5).

It has been shown that *H. volcanii* responds to changes in environmental salinity by modulating the *N*-linked glycans decorating the S-layer glycoprotein [6], with a second *N*-glycosylation pathway being recruited in cells grown at lower salt concentrations [19]. Expression of these alternative glycosylation pathway genes results in a glycan chain that is different in composition than that produced by the main glycosylation pathway. The alternative glycosylation pathway in *H. volcanii* is composed of genes *agl5—agl15* which have been shown to participate in the generation of the low-salt tetrasaccharide [21]. Using BLASTX [57], we found that most of the genes comprising the alternative pathway in *H. volcanii* are absent in the isolates. We compared putative alternative glycosylation clusters in the *Haloferax* genomes described above, based on the presence of the hallmark gene *agl6* [21], which is present in all isolates except 24N and 47N. From preliminary analysis we discovered that in all the isolate clusters there was an adjacent *pilA2* gene downstream to *agl6*. PilA2 was conserved in all of the isolates, including 24N and 47N, which upon further examination also had multiple glycosylation and flippase-related genes adjacent to *pilA2*, as in the rest of the isolates. In contrast to the major glycosylation pathway (Figure 2), the alternative glycosylation clusters were highly variable across the different genomes (Figure 5).

Although isolate 6N putatively encodes a major glycosylation pathway homologous to that predicted in *H. gibbonsii*, examination of the alternative pathway revealed that glycosylation genes from this isolate are more related to their *H. volcanii* counterparts. Most of the isolates also have an Orc1/CDC6-encoding gene, usually involved in origin recognition during replication initiation, indicating that these genes and their neighboring clusters could originate from replicating mobile elements, such as plasmids. Also, most isolates encode a putative glycosyltransferase which is homologous to an uncharacterized gene (*HVO_2043*) adjacent to the alternative glycosylation pathway gene cluster in *H. volcanii.*

Isolates from the *H. gibbonsii* clade and *H. volcanii* also share a gene encoding a free-standing endonuclease with a LAGLIDADG motif family 2 motif (pfam03161). Indeed, its flanking DNA does not have significant similarity to any protein-coding gene, confirmed by a sensitive BLASTX [57] search. Such homing endonuclease-encoding genes tend to be found in introns and inteins within the open reading frames of other genes. However, free standing homing endonuclease were previously described, mostly in bacteriophages [63,64]. Isolates 24N and 47N do not have *agl6* homologs, however they do contain type IV pilin homologs with adjacent glycosylation-related genes, as in the other clusters.

Phylogenetic analysis of the glycosyltransferase-encoding *agl6* gene and its homologs in the isolates and isolate-related species revealed a different tree from the conserved *rpoB1* tree as seen in Figure 4d. The *agl6* tree still has two main clusters: the *H. volcanii* clade and the *H. gibbonsii* clade. However, the *H. gibbonsii* clade, in contrast to the *H. volcanii* clade, differs notably from the conserved *rpoB1* phylogeny. *H. gibbonsii* diverges from isolates 4N and 6N contrary to the *rpoB1* tree where they cluster together.

## 4. Discussion

Here, we show that even a population of strains isolated from two sampling sites less than 40 km apart can present considerable diversity in terms of cell surface biology and the genes involved. Our data demonstrate that genes within a clade of S-layer glycoproteins and *N*-glycosylation genes can be conserved and show traces of recombination of some of those genes that mostly involve other members of the same clade. Such small scale recombination events have been shown to be common in halophilic archaea, especially within lineages [51]. This pattern of preferred recombination within lineages that results in diversification is in agreement with previous work showing that biased HGT can contribute to speciation processes as much as vertical descent. Similar observations have been also been made in bacteria [65,66,67].

Viruses may be one of the key drivers of within-species genome diversity in prokaryotes and this diversity often takes the form of genomic islands that contain genes present in a only small subset of strains that directly affect host-virus interactions [68]. In cyanobacteria, genomic islands encode surface factors that confer susceptibility or resistance to different bacteriophages [69]. In the haloarchaeon *Haloquadratum walsbyi* DSM 16790, two different genomic islands encode glycosyltransferases and S-layer glycoproteins [70]. Thus, viruses may exert an influence that is comparable to host immune system, producing not just different surface types, analogous to serotypes in bacterial pathogens but also a mechanism of switching the from one surface type to another as often seen in human pathogens and commensals [71,72].

In contrast to the major glycosylation clusters, alternative glycosylation clusters appear highly variable in gene content, even within the two *Haloferax* clades. Curiously, that cluster has a G + C content of 52%, much lower than any of the *Haloferax* genomes (Table 1), which is typical to mobile genetic elements that tend to have lower G + C than their hosts [73]. Furthermore, many of these clusters have an *orc1* gene, implying that they are derived from self-replicating mobile elements such as plasmids. The high level of variation in these clusters may give rise to different glycosylation patterns. Given that variation of the cell surface, such as a change of S-layer glycoprotein glycosylation pattern, could help achieve resistance against viral elements that bind to sugar receptors, such alternative clusters could provide a form of anti-viral defense, if expressed when cells become infected. Indeed, the alternative glycosylation cluster of *H. volcanii* is expressed under low salt conditions, which also induce expression of CRISPR, a veritable anti-viral defense, in *H. mediterranei* [74]. Thus, low-salt exposure for halophiles could induce similar envelope stress as associated with virus contact or entry, a stress that is thought to induce a CRISPR-Cas-mediated defense in bacteria [75]. Curiously, under low salt conditions, both wild type and *ΔaglB H. volcanii* cells become non-motile and form microcolonies [3], perhaps adapting a structure that is less sensitive to viral pressure, or less conducive for viral spread.

The pilin gene adjacent to the alternative glycosylation locus in *H. volcanii*—known as *pilA2*—has a G + C content of 58.7%, which is markedly lower than the genomic average, yet much higher than that of the glycosylation locus (see above). Interestingly, PilA2, which is one of the most highly expressed pilins in *H. volcanii* is highly *N*-glycosylated by AglB under optimal salt conditions [3] but whether under low salt, when AglB-mediated glycosylation is highly reduced, it undergoes glycosylation by the alternative pathway, remains to be determined.

Notably, most alternative glycosylation clusters in our isolates also contain free-standing homing endonuclease genes (i.e., homing endonucleases that are not located within an intron or an intein). Such enzymes typically recognize a highly specific target site over 20 bases long that appears only once in a typical archaeal genome and once they invade that site, the genome becomes immune to further cleavage [76]. This raises an intriguing possibility, namely that parts of these islands can sometimes be transferred via gene conversion, where the break is initiated by the homing endonuclease. Previous work has shown that homing in *Haloferax* can result in gene conversion events larger than 60 kb [77]. Thus, in principle, the whole island could be transferred in this fashion, when a target site exists in the recipient. Thus far, such free-standing homing endonucleases have mostly been observed in bacteriophages, where they cut the DNA of specific competing phages during co-infection [64], a process that can lead either to repair (and thereby invasion of the other phage by homing) or to the degradation of the viral DNA. Whether these endonucleases in haloarchaeal genomes can also provide resistance against viruses or other mobile genetic elements requires further study.

## 5. Conclusions

Here, we describe a new type of S-layer glycoprotein found in multiple *Haloferax* strains and provide evidence for within- and between-lineage gene transfer in the major and alternative glycosylation clusters in *Haloferax* that may result in situations where no two isolates present an identical surface profile. Such within-niche variation is expected to enhance the resistance of the *Haloferax* meta-population to viral pressures and may generate patterns of mating preference.

## Figures and Tables

**Table 1:** List of *Haloferax* isolates collected from four pools from two rocky shores along Israel’s Mediterranean coastline (Atlit and Michmoret) and related *Haloferax* species added to the analysis due to their similarities to the isolates. * Total length includes all replicons.

Isolate Number	Place of Isolation	Number of Contigs over 1 Kbp	GC Content (%)	Total Length (bp) *
4N	Michmoret pool1	32	65.31	4,196,082
6N	Michmoret pool2	23	65.12	4,414,718
10N	Michmoret pool2	54	65.2	4,278,547
12N	Michmoret pool2	46	65.32	4,031,013
16N	Michmoret pool2	10	65.74	3,971,935
19N	Atlit pool2	29	64.29	4,185,404
24N	Atlit pool4	9	65.74	3,914,077
47N	Atlit pool8	11	65.84	3,845,131
48N	Atlit pool8	11	65.82	3,925,572
*Haloferax volcanii* DS2	Dead Sea		65	4,012,900
*Haloferax gibbonsii* ATCC 33959	Saltern, Rio de Janeiro		62.8	3,918,454
*Haloferax denitrificans* ATCC 35960	Saltern, California		66.3	3,825,970
*Haloferax lucentense* DSM 14919	Saltern, Spain		66.39	3,619,064

## Supplementary Materials

The following are available online at http://www.mdpi.com/2073-4425/9/3/172/s1, Figure S1: Recombination events in the *rpoB1* sequence, Figure S2: Recombination events in the SLG sequence of the *H. gibbonsii* clade, Figure S3: Recombination events in the SLG sequence of the *H. volcanii* clade, Figure S4: Recombination events in the *aglJ* sequence, Figure S5: Recombination events in the *aglD* sequence, Figure S6: Recombination events in the *aglB* sequence, Figure S7: SDS-PAGE gel of the isolates and *H. volcanii* DS2, Figure S8: Sequence alignment of the homing endonuclease with the LAGLIDAG motif seen in the alternative glycosylation pathway, Table S1: Matrix of average nucleotide identity (ANI) between the isolates, *H. volcanii*, *H. gibbonsii*, *H. lucentense* and *H. denitrificans*, Table S2: GC content of all genes mentioned before in the isolates and the related *Haloferax* species.
