# Supplementary material for: Comparative Analysis of Surface Layer Glycoproteins and Genes Involved in Protein Glycosylation in the Genus Haloferax
Source: Genes (Basel). 2018 Mar 20;9(3):172. doi: 10.3390/genes9030172 (PMC5867893; doi:10.3390/genes9030172)
Supplement: Supplementary file 1 [file genes-09-00172-s001.docx]

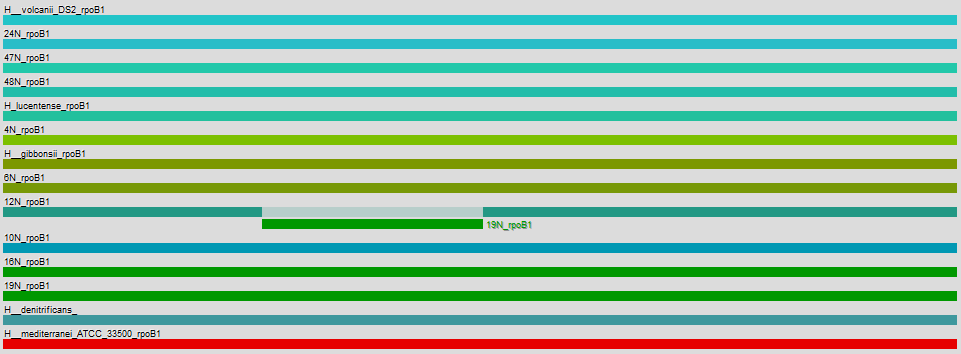


**Figure S1.** Recombination events in the *rpoB1* sequence. The one recombination event found in isolate 12N in positions 496-920 with 19N as the major parent is supported only by the RDP method and the SiScan algorithm, so was disregarded.


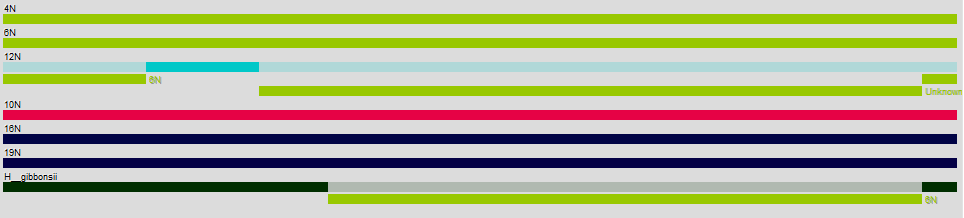


**Figure S2.** Recombination events in the SLG sequence of the *H. gibbonsii* clade. One recombination event occurs in *H. gibbonsii* (position 936-2648 in the SLG sequence) and two occur in 12N (positions 413-3647 and 736-2647 in the SLG sequence), all events list 19N as the major parent and all events are supported by all methods.


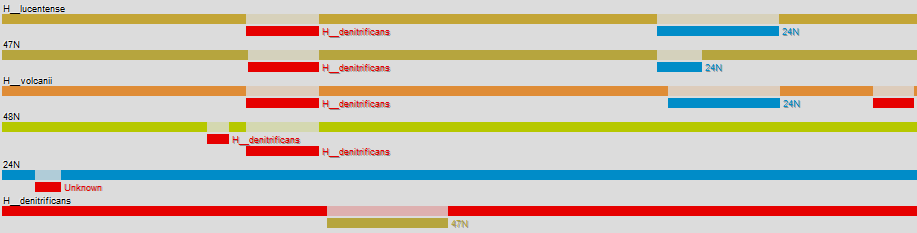


**Figure S3.** Recombination events in the SLG sequence of the *H. volcanii* clade. In the *H. volcanii* clade there are 3 detected recombination events. The first is detected in *H. volcanii, H. lucentense* and 47N in position 1842-2152 of the SLG nucleotide sequence, the proposed major parent is 48N and is supported by all the methods. The second recombination event is detected in *H. volcanii* in position 2408-2522 the proposed major parent is 48N and is supported by all the methods as well. The third event occurs in position 92-164 in isolate 24N. The proposed major parent is isolate 47N and this event is supported by all the methods except SiScan.


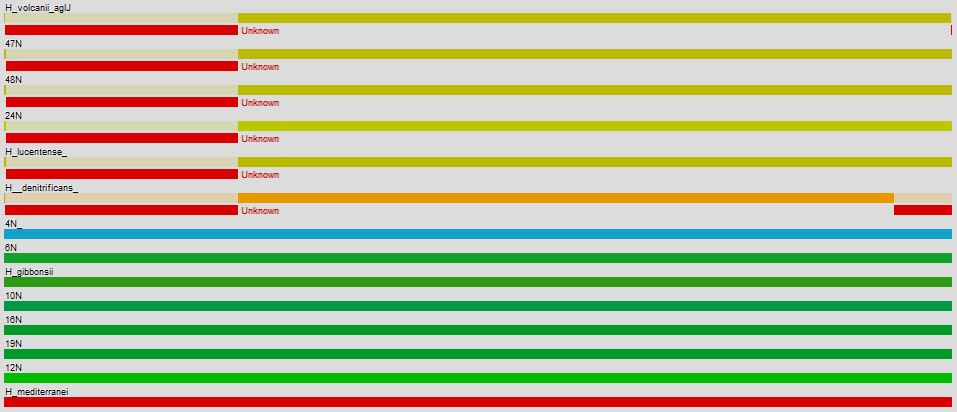


**Figure S4.** Recombination events in the *aglJ* sequence. One recombination event was found in the *H. volcanii* clade in position 1-222 of the *aglJ* sequence and supported by all methods. The proposed major parent is 12N.


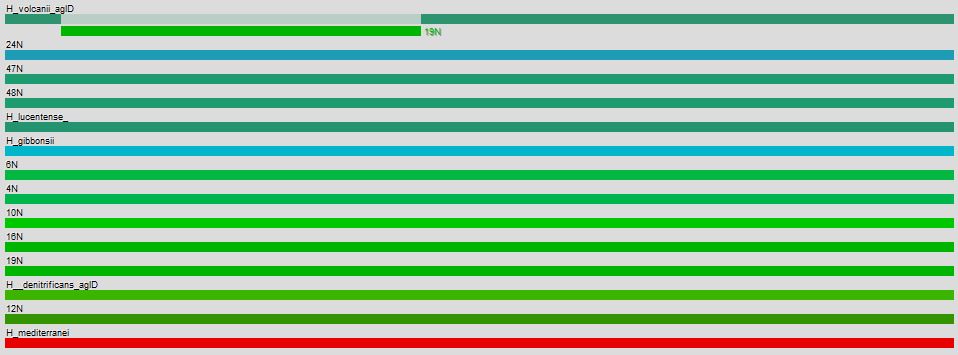


**Figure S5.** Recombination events in the *aglD* sequence. One recombination event was found in *H. volcanii* in positions 111-822. Suspected major parent is isolate 48N and the event is supported by all methods.


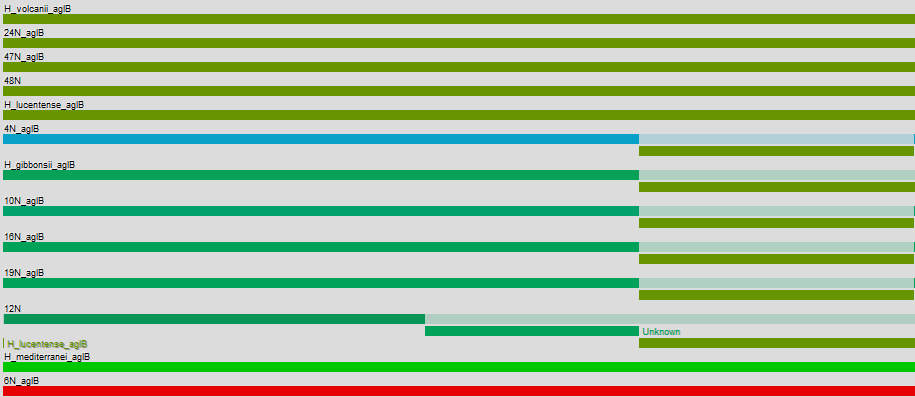


**Figure S6.** Recombination events in the *aglB* sequence. The two events both occur in the *H. gibbonsii* clade. The first event in positions 2427-3478 of the *aglB* sequence is supported by all methods and is found in 4N, 10N, 12N, 16N, 19N and *H. gibbonsii*. The proposed major parent is *H. mediterranei***.** The second recombination event is found only in 12N with *H. gibbonsii* being the proposed major parent and supported by all the models except GENECONV and BootScan.

**
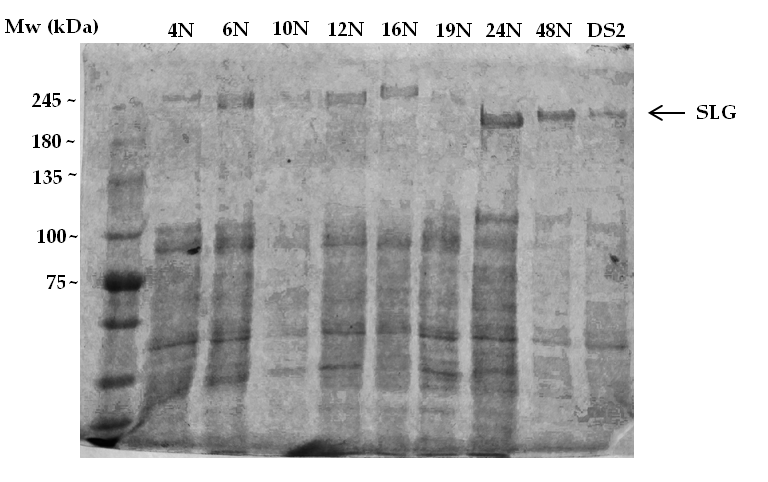
**

**Figure S7.** SDS-PAGE gel of the isolates and *H. volcanii* DS2**.**

**
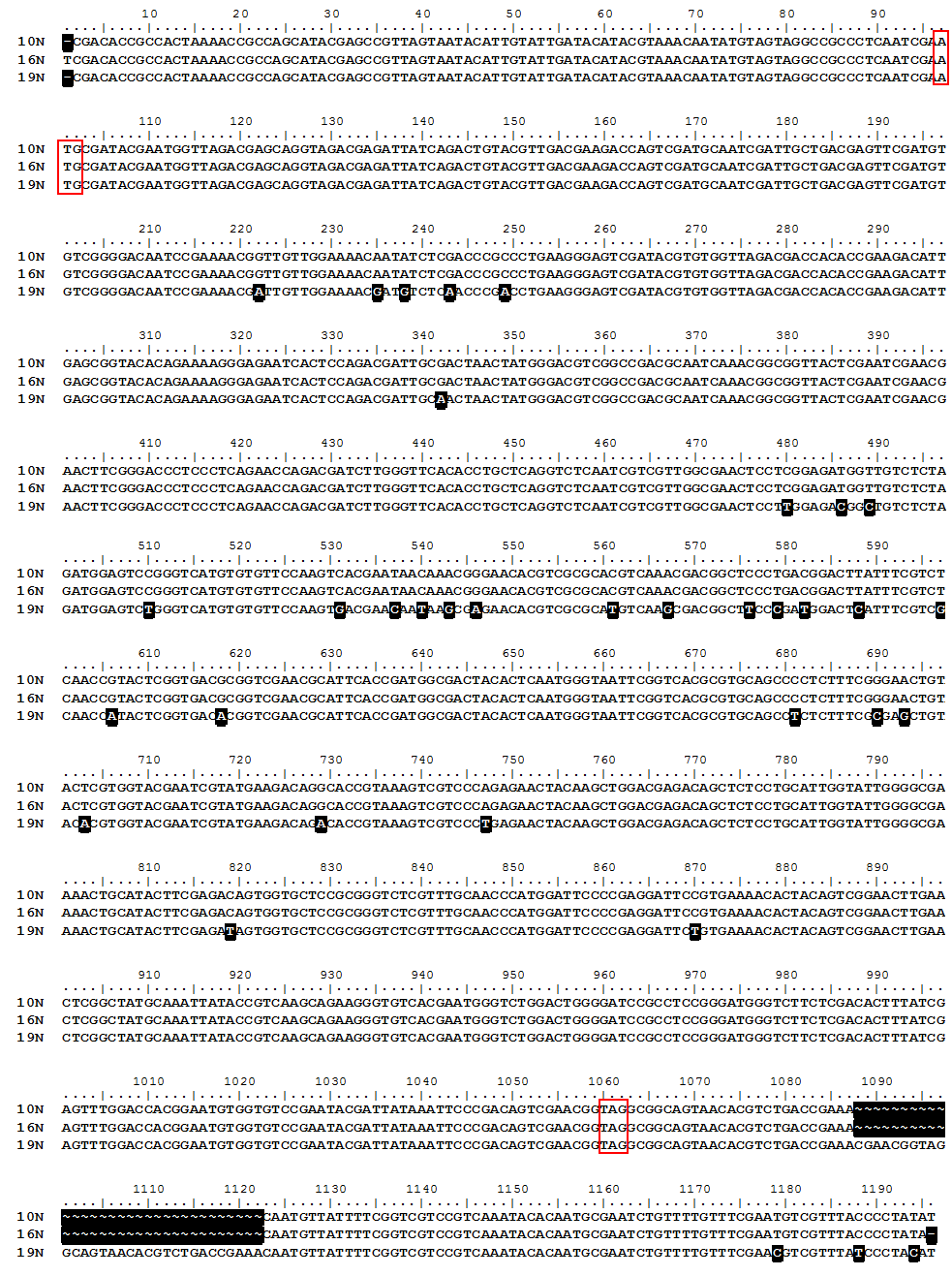
**

**Figure S8.** Sequence alignment of the homing endonuclease with the LAGLIDAG motif seen in the alternative glycosylation pathway. The protein encoding this endonuclease is identical in isolates 10N and 16N and they are 99% identical to 19N. Sequences were aligned using [78]. Start and stop codons are marked in red boxes.

**Table S1.** Matrix of average nucleotide identity (ANI) between the isolates, H. volcanii, H. gibbonsii, H. lucentense and H. denitrificans. Order of the species and isolates is by degree of identity. Isolates and species grouped together in purple boxes have a high average of nucleotide identity and are possibly strains of the same species. ANI was calculated using the Kostas Konstantinidis’ lab website (http://enve-omics.ce.gatech.edu/ani/).

| ***aglJ*** | ***aglB*** | ***rpoB1*** | ***aglD*** | ***agl6*** | **SLG** | **Genome** |  |
| --- | --- | --- | --- | --- | --- | --- | --- |
| 60.60% | 62.50% | 66.39% | 70.56% | 62.24% | 61.88% | 65.00% | ***H. volcanii*** |
| 60.49% | 62.46% | 66.45% | 70.61% | - | 61.55% | 65.84% | **47N** |
| 60.49% | 62.46% | 66.45% | 70.61% | 61.46% | 61.35% | 65.82% | **48N** |
| 60.60% | 62.50% | 66.50% | 70.61% | - | 59.45% | 65.74% | **24N** |
| 60.60% | 62.40% | 66.28% | 70.51% | 62.00% | 61.79% | 66.39% | ***H. lucentense*** |
| 59.82% | 62.00% | 66.17% | 70.93% | - | 59.66% | 66.30% | ***H. denitrificans*** |
| 65.48% | 66.09% | 66.72% | 70.93% | 60.88% | 61.92% | 65.31% | **4N** |
| 65.48% | 66.06% | 66.72% | 71.04% | 60.24% | 61.92% | 65.12% | **6N** |
| 65.15% | 66.12% | 66.61% | 71.04% | 60.16% | 62.40% | 62.80% | ***H. gibbonsii*** |
| 65.37% | 65.36% | 65.90% | 70.40% | 60.88% | 62.47% | 65.20% | **10N** |
| 65.37% | 65.36% | 65.90% | 70.40% | 60.88% | 62.47% | 65.74% | **16N** |
| 65.37% | 65.39% | 65.90% | 70.40% | 61.37% | 62.29% | 64.29% | **19N** |
| 65.48% | 65.81% | 65.90% | 69.97% | 61.20% | 61.87% | 65.32% | **12N** |

Table S2. GC content of all genes mentioned before in the isolates and the related *Haloferax* species.

References

1. Hall, T.A. BioEdit: A user-friendly biological sequence alignment editor and analysis program for Windows 95/98/NT. *Nucleic Acids Symp. Ser.* **1999**, *41*, 95–98.
